# Portal Vein Thrombosis in a Patient With Methylenetetrahydrofolate Reductase Gene Mutation and Normal Homocysteine Levels

**DOI:** 10.7759/cureus.18225

**Published:** 2021-09-23

**Authors:** Ghulam Mujtaba Ghumman, Rizwan Ishtiaq, Deepti Avasthi

**Affiliations:** 1 Internal Medicine, St. Vincent Mercy Medical Center, Toledo, USA

**Keywords:** anticoagulation, homocysteine, methylenetetrahydrofolate reductase, thromboembolism, portal vein thrombosis

## Abstract

A 21-year-old male presented with chief complaints of abdominal pain, nausea, and vomiting and was found to have portal vein thrombosis (PVT) on computed tomography (CT) scan of the abdomen, which was redemonstrated on ultrasound. Thrombophilia workup was negative except that patient was heterozygous for methylenetetrahydrofolate reductase (MTHFR) gene mutation. Homocysteine levels were normal. The patient was started on enoxaparin and discharged on apixaban with the plan to continue anticoagulation for at least six months. Follow-up MRI after four months showed interval improvement of the main portal vein thrombus with the use of Eliquis.

## Introduction

Portal vein thrombosis (PVT) is an occlusion of the portal vein with or without extension to the splanchnic venous system. PVT can be acute or chronic, and accurate management of PVT depends on the underlying cause identified [1]. Entities that can cause PVT include cirrhosis or tumoral invasion, or extrinsic compression from a tumor. Close to 30% of the patients with PVT have no cause identified, and these cases are labeled as idiopathic PVT [2]. Familiarity with the disease presentation and risk factors is vital to diagnose the condition accurately and timely for appropriate management. In this article, we describe a case of a 21-year-old male who presented with a chief complaint of abdominal pain and was found to have PVT secondary to a heterozygous mutation in the methylenetetrahydrofolate reductase (MTHFR) gene with normal homocysteine levels.

## Case presentation

A 21-year-old Caucasian male presented to the emergency department (ED) with chief complaints of nausea, vomiting, and abdominal pain for three days. He described the abdominal pain as sharp, band-like, and 10/10 in intensity. He stated that his pain is somewhat alleviated with hot showers and baths, and aggravated with movement and sleeping on his side. One day prior, he was seen at urgent care for the same complaints, where he was prescribed dicyclomine and ondansetron and was discharged. He reported no improvement in his symptoms with these medications. The patient denied any complaints of fever, chills, diarrhea, hematuria, or rectal bleeding. His past medical history was insignificant, and he was not taking any medicines regularly. The patient denied any history of smoking, alcohol use, or recreational drug use. Family history was insignificant for any clotting or gastrointestinal disorder. On physical examination, the patient appeared to be in mild distress from abdominal pain. His hemodynamics were stable. The abdomen was soft, non-distended with tenderness in the right and left lower quadrants without any guarding. Bowel sounds were audible and regular. There were no clinical signs of liver cirrhosis, including but not limited to ascites, spider angioma, or palmar erythema. The rest of the examination was unremarkable.

**Figure 1:**
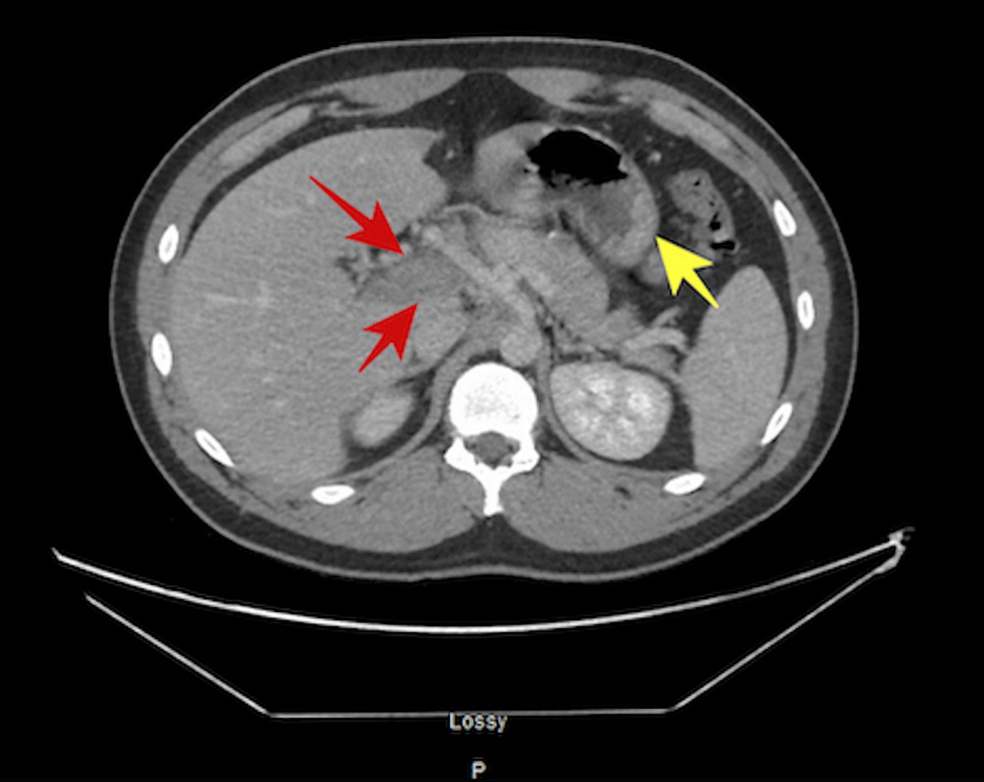
Axial CT scan of the abdomen shows an area of low attenuation in the region of portal vein suggestive of portal vein thrombosis (red arrows) and submucosal fat attenuation and signs of inflammation in the visualized portion of the colon suggestive of burned-out colitis or inflammatory bowel disease (yellow arrow).

**Figure 2:**
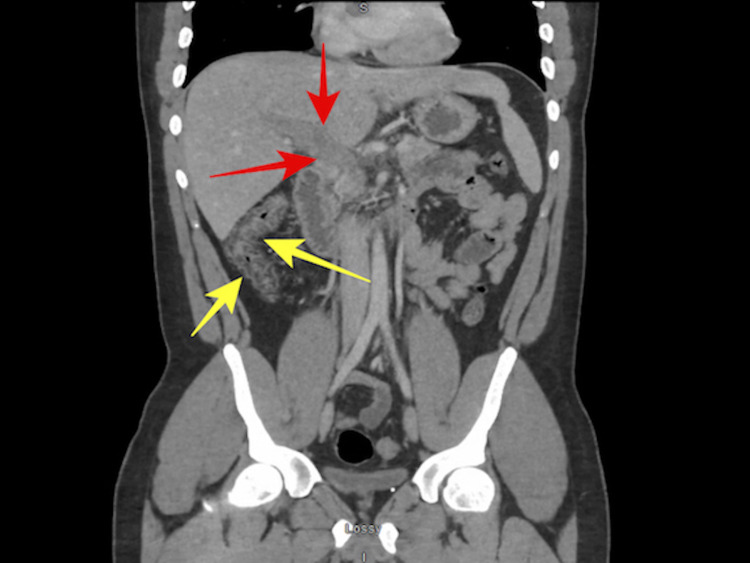
Coronal CT scan of the abdomen and pelvis shows an area of low attenuation in the region of portal vein suggestive of portal vein thrombosis (red arrows) and submucosal fat attenuation and signs of inflammation in the visualized portion of the colon suggestive of burned-out colitis or inflammatory bowel disease (yellow arrow).

His initial laboratory workup revealed elevated leukocyte count at 15.3 k/uL, elevated alanine transaminase (ALT) level at 59 U/L, and elevated total bilirubin at 1.48 mg/dl with indirect bilirubin of 1.12 mg/dl and direct bilirubin of 0.36 mg/dl, while rest of the complete blood count (CBC) and comprehensive metabolic panel (CMP) was within normal range. The hepatitis profile was unremarkable, and the lipase level was within the normal range. Computed tomography (CT) scan of the abdomen and pelvis with contrast, ordered to look for any obstruction or malignancy, revealed non-specific hypo-attenuation in the regions of the portal vein and portal splenic confluence suggestive of portal vein thrombosis (Figures 1, 2). A small amount of free fluid in the pelvis and submucosal fat attenuation throughout the colonic segments was also appreciated, which can be seen with the history of burned-out colitis, inflammatory bowel disease (IBD), acute ischemia of the bowel secondary to clot in mesenteric arteries, or as a normal finding in obese patients. The suspicion of PVT was confirmed with the ultrasound imaging of the liver.

The patient's IBD panel was not consistent with inflammatory bowel disease. Calprotectin levels were normal. The patient was referred for colonoscopy, but the gastroenterology team did not feel the need for colonoscopy at that point. Hypercoagulable workup, which was done before starting anticoagulation, was grossly in normal range with an international normalized ratio (INR) of 1.0, protein C activity was mildly low at 78% (normal reference range: >80%), while protein C total antigen was normal at 94% (normal reference range: 63-153%). Protein S functional assay and protein S free antigen were normal at 89% (normal reference range: 77-116%) and 146% (normal reference range: 74-147%), respectively, while protein S total antigen was mildly elevated at 154% (normal reference range: 84-134%). Factor 5 assay, prothrombin 20210 mutation, anti-thrombin III activity and antigen, factor 2 assay, and lupus anticoagulant were all negative. D-dimer levels were not checked. Molecular testing revealed that the patient was heterozygous for the MTHFR A1298 mutation and negative for the MTHFR 677T mutation. Homocysteine level was normal at 8.5 (normal reference range: <15 umol/l). Janus kinase 2 (JAK-2) mutation was not checked as the rest of the laboratory values were not suggestive of any myeloproliferative disorder.

The patient was started on full-dose enoxaparin for anticoagulation and was discharged on apixaban with the plan to continue for at least six months. Follow-up magnetic resonance imaging (MRI) of the abdomen revealed remaining/residual chronic thrombus of the main portal vein, right portal vein, and superior mesenteric vein at the portal vein confluence. However, there has been interval improvement of the main portal vein thrombus with the use of Eliquis over four months.

## Discussion

There is a wide range of etiologies responsible for causing PVT. A prothrombotic factor can be found in 70% of the patients with PVT. Systemic factors are found in approximately 60% of the patients, and these factors include the presence of any myeloproliferative neoplasm, antiphospholipid syndrome, paroxysmal nocturnal hemoglobinuria, hereditary thrombophilia factor deficiencies, or mutations like MTHFR and prothrombin gene mutation [3-4]. The most common cause for the development of PVT is the presence of myeloproliferative neoplasms [3-4]. Pregnancy and the use of oral contraceptives have also been linked to the development of PVT [3]. No cause for the development of PVT (idiopathic PVT) is identified in almost 30% of the patients [3-4].

Clinical manifestations of PVT depend mainly on the acuity of disease presentation [1]. The acute phase usually presents with abdominal pain, fever, malaise, nausea, and postprandial fullness, and can have a transient elevation of liver enzymes [1-4]. In contrast, patients with chronic PVT have a normal liver function in general, and it is usually an incidental finding during radiological or endoscopic studies in patients with signs of portal hypertension [1-3]. Ultrasonography is the imaging of choice for diagnosis of PVT, while a CT scan or MRI of the abdomen can be performed later to confirm the diagnosis [5-6]. In our case, the patient presented with abdominal pain with elevation of liver enzymes and absence of ascites, which correspond to the development of PVT in an acute phase.

**Figure 3:**
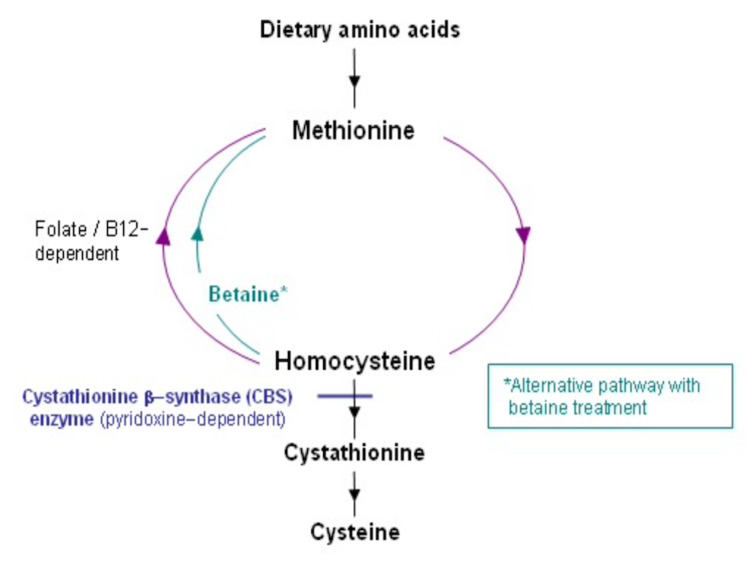
Metabolism of homocysteine.

Our discussion mainly focuses on the MTHFR gene mutation as a potential cause of deep vein thrombophilia, including PVT. MTHFR gene encodes the MTHFR enzyme, which is a key regulatory enzyme in folate and homocysteine metabolism [7]. Homocysteine is an amino acid that is metabolized by either cystathionine beta-synthase (which requires vitamin B6) or methionine synthase (which requires folate and vitamin B12) to cystathionine or methionine, respectively (Figure 3) [8].

Deficiency of any of the enzymes or their cofactors in the homocysteine metabolism can lead to elevated homocysteine levels in the blood (hyperhomocysteinemia) [9]. MTHFR enzyme catalyzes the conversion of methylenetetrahydrofolate to methyl-tetrahydrofolate, which is used for methylation of homocysteine to methionine [7]. Mutations in the MTHFR gene lead to impaired functioning or inactivation of the MTHFR enzyme, which can disrupt the homocysteine metabolism leading to elevation of homocysteine levels in the blood [10]. Elevated homocysteine level is associated with an increased risk of venous thromboembolism and atherosclerotic diseases of the heart and brain [11]. The most common MTHFR polymorphisms are C677T and A1298C, which can be either homozygous or heterozygous [12]. MTHFR deficiency can lead to rare hyperhomocysteinemia with homocystinuria, or more commonly mild to moderate elevation in homocysteine levels [13]. The severe form of hyperhomocysteinemia with homocystinuria has been observed with 34 different MTHFR gene mutations, while the milder form has been studied to be associated with the C677T variant that encodes a thermolabile MTHFR enzyme with reduced activity [12]. A recent study showed that the heterozygous or homozygous A1298C variant does not increase the level of homocysteine [10], as also observed in our case.

Portal vein thrombosis is rare but has been observed in patients with MTHFR mutations and most of the cases of thromboembolism reported in MTHFR mutation occurred in the setting of hyperhomocysteinemia [14]. Although it appears from the above discussion that elevated homocysteine level is the main reason behind the development of venous thromboembolism in patients with MTHFR mutation, many studies have shown that there is an increased risk of venous thromboembolism in patients with MTHFR mutations and normal homocysteine levels, as also seen in our case. A retrospective analysis published in the year 2009 in the *Journal of Clinical Oncology* showed that 92% of patients with a documented vascular event (deep vein thrombosis, pulmonary embolism, myocardial infarction, or cerebrovascular accident) had MTHFR but normal homocysteine levels with no other significant risk factors that could explain thrombophilia [15]. Another case-control study found that mutations in C677T and 1298C alleles led to an increased tendency to develop coronary artery disease even with normal homocysteine levels [16]. A rare case has been described by Gürsoy et al. where the patient had combined splenic, portal, and mesenteric vein thrombosis associated with heterozygous mutation of MTHFR gene but with normal homocysteine levels, although there was an acquired factor of ulcerative colitis in their case, which can be an independent risk factor for thrombosis [17]. It has also been observed that the risk of thromboembolism did not improve after correcting homocysteine levels (in those patients with MTHFR gene mutation who had hyperhomocysteinemia), suggesting that an alternate mechanism for the occurrence of thromboembolism might exist [18]. The similarity between the cases mentioned above and our case is the development of portal vein thrombosis in patients with MTHFR mutation with normal homocysteine levels. Current guidelines do not advise screening patients for MTHFR mutations; instead, screening only for homocysteine levels to evaluate the risk for thromboembolism [19]. These mutations can be missed in patients with normal homocysteine levels and no other identifiable cause of thrombophilia if they are not screened for genetic mutations.

The mainstay of treatment for PVT is prompt initiation of anticoagulation therapy unless contraindicated. Recanalization of the portal vein prevents the development of portal hypertension, which has an impact on prognosis [20]. Anticoagulation should be continued for at least six months, as recanalization of the portal vein can take up to six months to occur. Recanalization of the splenic and mesenteric veins can take up to one year, so involvement of these veins is an indication for continuing anticoagulation for up to one year [4]. Patients with thrombophilic disorder and personal or family history of venous thrombosis require lifelong anticoagulation [20].

## Conclusions

Overall, the studies investigating the association of MTHFR mutations and venous blood clots have been inconsistent, with some studies showing a slight association, but most studies have not shown any association. Over the past 15 years, several studies have looked at MTHFR mutations and the risk of various disorders. More than 615 medical disorders had been researched, with most of the work relating to homocysteine levels, thrombosis, cardiovascular disease risk, cancer risk, neural tube defects, pregnancy complications, and psychiatric disease. To date, the studies have been conflicting, with some showing that MTHFR mutations are related to these additional disorders, whereas others show no association. Often, the results have depended on the ethnicity and geographic location of the population studied, indicating that the results may be influenced by various confounding factors, including the multifactorial nature of the disorders and inability to identify the multiple genetic and environmental factors that interact with MTHFR polymorphisms to impact disease risk. The observance of thrombophilia with normal homocysteine levels and the fact that correcting homocysteine levels in patients with hyperhomocysteinemia does not decrease the risk of thromboembolism suggests that MTHFR mutation independently can lead to thromboembolism without hyperhomocysteinemia. Therefore, it is recommended that patients with thrombophilia with no other apparent identifiable factor need to have screening for genetic mutations. Animal studies designed to study the tissue-specific impact of MTHFR mutation may be helpful to get more knowledge about the disease and manage the complications properly.
